# Spermidine Remodels the Mitochondrial Metabolism of Tumor‐Infiltrating Lymphocytes

**DOI:** 10.1155/jimr/7550012

**Published:** 2025-10-29

**Authors:** Yizhe Sun, Hao Fu, Xinyu Li, Meng Wan, Hongchao Xiong, Chaoting Zhang

**Affiliations:** ^1^ Key Laboratory of Carcinogenesis and Translational Research (Ministry of Education/Beijing), Laboratory of Biochemistry and Molecular Biology, Peking University Cancer Hospital and Institute, Beijing, China, pku.edu.cn; ^2^ Division of Pathology, Department of Laboratory Medicine, Karolinska Institutet, Stockholm, Sweden, ki.se; ^3^ The First Department of Thoracic Surgery, Peking University Cancer Hospital and Institute, Beijing, China, pku.edu.cn; ^4^ Department of Radiation Oncology, Beijing Obstetrics and Gynecology Hospital, Capital Medical University, Beijing, China, ccmu.edu.cn

**Keywords:** metabolism, mitochondria, spermidine, TCA cycle

## Abstract

Adoptive cell therapy (ACT) utilizing tumor‐infiltrating lymphocytes (TILs) has significant potential in treating various cancers; however, its effectiveness is often compromised by the tendency of TILs to become exhausted and dysfunctional. Revitalizing these essential immune cells is crucial for amplifying their antitumor efficacy. Our study investigates the influence of spermidine on the metabolic pathways of TILs, focusing on its critical contribution to T cell vitality. We assessed the impact of spermidine on glucose absorption, mitochondrial functionality, and energy production in TILs. The application of spermidine resulted in a pronounced improvement in mitochondrial functionality and energy production, indicated by a surge in mitochondrial numbers and enhanced activity of the tricarboxylic acid (TCA) cycle. Importantly, the suppression of mitochondrial metabolism negated the beneficial effects of spermidine on mitigating exhaustion and enhancing cellular activity, highlighting the essential role of mitochondrial metabolism in the action of spermidine. Our research suggests that modulation of metabolism by spermidine could be a potential strategy to strengthen the antitumor capabilities of TIL–based treatments, offering a promising method to better manage solid tumors.

## 1. Introduction

Adoptive cell therapy (ACT) utilizing tumor‐infiltrating lymphocytes (TILs) has demonstrated efficacious antitumor activities across a variety of oncological setting [1–3]. However, the therapeutic efficacy of TILs is notably compromised by T cell exhaustion, a state characterized by diminished proliferative abilities, the upregulation of inhibitory immunoreceptors, and a reduced capacity to produce cytotoxic effector molecules [4].This highlights the imperative for in‐depth exploration into the attributes of T cell exhaustion within TILs and the development of strategies aimed at ameliorating this exhausted state to augment the antitumor efficacy of TIL–based therapies.

The relationship between T cell metabolism and T cell exhaustion represents a critical area of investigation within the field of immunology, shedding light on the mechanisms that underlie the dysfunction of T cells during chronic infections and cancer [4, 5]. A growing body of evidence underscores the pivotal role of metabolic processes in governing T cell fate and function. Upon activation, T cells undergo a metabolic reprograming from oxidative phosphorylation (OXPHOS) to aerobic glycolysis, a shift that supports the biosynthetic and energetic demands of cell proliferation and effector function [6]. However, in the context of chronic antigen exposure, as seen in cancer and chronic viral infections, T cells exhibit impaired metabolic fitness, characterized by a diminished capacity to uptake and utilize nutrients, which contributes to the development of T cell exhaustion [7]. Metabolic reprograming in exhausted T cells is not merely a consequence of chronic stimulation, but plays a proactive role in the exhaustion process. Exhausted T cells exhibit altered signaling pathways that directly impact metabolic processes, including decreased PI3K/Akt/mTOR signaling, which is crucial for glycolysis and cellular growth [8]. Moreover, the upregulation of inhibitory receptors, such as PD‐1, has been linked to metabolic dysregulation in T cells. Engagement of PD‐1 with its ligand PD‐L1 on tumor cells leads to the inhibition of Akt/mTOR signaling, further impairing glycolysis and promoting T cell exhaustion [9].

Therefore, interventions aimed at modulating T cell metabolism have shown promise in reversing T cell exhaustion and restoring antitumor immunity. For instance, treatments that enhance glycolysis or augment mitochondrial biogenesis and function can improve the effector functions of exhausted T cells, suggesting that metabolism‐aiming therapies could represent a novel avenue for cancer immunotherapy [10].

Spermidine, a polyamine found in various foods, has gained attention for its role in enhancing cell function, particularly through the promotion of autophagy, The amelioration of hepatocellular fibrosis and carcinogenesis mediated by autophagy induction via spermidine has been proven [11]. Similar effect on reversal of dysfunction of cells has been revealed in cardiovascular system and lung tissue [12–14]. This effect is also crucial for cellular cleaning and renewal, which supports the health and efficiency of immune cells. A study has demonstrated that spermidine may reverse B cell aging by activating eIF5A hypusination and subsequently inducing autophagy via TFEB [15]. In addition to regulating autophagy through hypusination of the TFEB pathway, spermidine also induces autophagy by suppressing the acetylation of histones and other proteins [16]. Modulation of lipid metabolism is demonstrated to be another way by which spermidine induces autophagy, and thus, affects the biological process [17, 18]. Studies have shown that spermidine supplementation can rejuvenate T cells, improving their proliferation and effector functions [19], thereby bolstering the immune response against infections and cancer [20, 21]. Such findings open the door for spermidine as a dietary supplement to potentially boost immune health and combat immunosenescence.

Increasing robust studies demonstrate spermidine rewire the T cells mitochondrial function by activation of trifunctional protein and enhance the anti‐tumor activity in vivo [22]. Also, given our previous study, we have proven spermidine can reverse the exhausted state of TILs and peripheral T cells from blood by enhancing autophagy, simultaneously, with the improvement of T cell functionality and cytotoxicity in vitro and in vivo [23, 24]. In our research, we demonstrate enhanced mitochondrial biogenesis and activity induced by spermidine. Moreover, inhibiting the mitochondrial pathway can nullify the effects of spermidine in reversing T cell exhaustion.

## 2. Methods and Materials

### 2.1. Isolation and Culture of TILs

This research received approval from the Institutional Review Board at Peking University School of Oncology, China. Lung cancer patients consented to the use of their cancer tissues for TIL extraction and subsequent analyses, as detailed in prior studies [25]. In summary, cancer tissues were sterilely minced into 1–2 mm fragments and placed in individual wells of a 24‐well plate filled with T cell culture medium. This medium comprised X‐VIVO 15 serum‐free medium (Lonza, USA), supplemented with GlutaMAX (Thermo Fisher, USA), IL‐2 (50 U/mL; PeproTech, USA), IL‐7 (10 ng/mL; PeproTech, USA), IL‐15 (10 ng/mL, PeproTech, USA), OKT3 antibody (50 ng/mL; ACRO, USA), and anti‐CD28 antibody (1 μg/mL; T&L Biotechnology, China). Cultured at 37°C in a 5% CO_2_ incubator, the TILs proliferated from the tissue pieces and were cryopreserved in liquid nitrogen upon reaching a density of about 1 × 10^6^ cells per well for later experiments.

For spermidine and oligomycin treatment, control group TILs were resuscitated and cultured in the X‐VIVO 15 serum with cytokines maintenance (IL‐2 [50 U/mL], IL‐7 [10 ng/mL], and IL‐15 [10 ng/mL]) for up to 7 days, while the experimental groups also received spermidine (10 μM; Cayman Chemical, USA) for the same duration. To assess the effect of the ATP synthase inhibitor oligomycin on spermidine‐treated TILs, TILs were exposed to spermidine for 7 days, with oligomycin (2 μM; Sigma–Aldrich, USA) added on the third day for an additional 5 days. To detect IFN‐γ production by flow cytometry, cytokines were withdrawn on Day 7, and the cells were maintained in a cytokine‐free medium for 1 day. OKT3 (50 ng/mL) or PMA (20 ng/mL) plus ionomycin (1 μg/mL) was then added for 12 h.

### 2.2. Cell Staining and Flow Cytometry

TILs were washed with phosphate‐buffered saline (PBS) and stained with fixable viability stain 780 (FVS780) in the dark at room temperature for 15 min. Membrane markers CD3, CD4, CD8, CD45, PD1, TIM3, and LAG3 (BD Biosciences, USA) were subsequently stained under similar conditions.

To assess intracellular markers, cells were initially fixed and permeabilized for 15 min at room temperature using the fixation and permeabilization solution provided by BD Biosciences. Following two washes with perm/wash buffer (also from BD Biosciences), cells were incubated with intracellular antibodies, specifically targeting Ki67 and IFN‐γ (both sourced from BD Biosciences, USA), in wash buffer for 15 min in the dark. Subsequent flow cytometric analysis was carried out after washing the cells twice with PBS. For the detection of intracellular IFN‐γ specifically, cells were pretreated with GolgiPlug, a protein transport inhibitor (BD Biosciences, USA), overnight. The recommended GolgiPlug usage was 1 µL per mL of medium (for ~1 × 10^6^ cells), ensuring thorough mixing.

For CD107a detection, cells were incubated overnight with a mixture of CD107a antibody and GolgiPlug, followed by flow cytometric analysis after staining for cell viability and other surface markers.

Analysis was performed on BD FACSAria and BD FACSCelesta SORP instruments, with data analyzed using FlowJo version 10.6.2 (BD Biosciences, USA).

### 2.3. DNA Extraction and Quantitative PCR (qPCR)

Following centrifugation and PBS washes, DNA was extracted using the DNeasy Blood and Tissue Kit (QIAGEN, Germany). qPCR was performed with TransStart Green qPCR SuperMix (TransGen Biotech, China), using 100 ng DNA templates and specific primers for mitochondrial DNA and the internal control human β‐globin. The PCR process was conducted on the StepOne Real‐time PCR System (Thermo Fisher, China).

### 2.4. Mitochondrial Assays

After 7 days of treatment with or without spermidine, cells were washed postcentrifugation and incubated with MitoTracker green FM (MTG) and MitoTracker orange (MTO) CMTMRos (Thermo Fisher, USA) probes in X‐VIVO 15 medium without additives for 15 min at 37°C in the dark. Cells were then washed with PBS and subjected to subsequent FVS780 and surface marker staining. Cells were incubated with 20 nM tetramethylrhodamine methyl ester (TMRM) at 37°C for 30 min for mitochondrial membrane potential (MMP) staining.

For the carbonyl cyanide m‐chlorophenyl hydrazone (CCCP)‐treated samples, CCCP (50 mM in DMSO, equilibrated to room temperature before use) was diluted 1:1000 into the suspension and incubated at 37°C with 5% CO_2_ for 5 min. For the oligomycin‐treated group, oligomycin was similarly diluted 1:1000 (2 μM) and incubated under the same conditions. Untreated samples were incubated in parallel. All groups were then stained with TMRM (20 μM in DMSO) at a final concentration of 20 nM and incubated for 30 min at 37°C in 5% CO_2_. Following incubation, samples were washed once with PBS (1400 rpm, 5 min) and resuspended in 500 μL of 1% BSA in PBS. Cells were subsequently analyzed by flow cytometry.

### 2.5. Glucose Intake Assay

Treated cells were incubated with 2‐[N‐(7‐nitrobenz‐2‐oxa‐1,3‐diazol‐4‐yl)amino]‐2‐deoxy‐D‐glucose (2‐NBDG; Thermo Fisher, USA) diluted in PBS, followed by a 2 h incubation at 37°C in the dark. Cells were then prepared for flow cytometry.

### 2.6. In Vitro Coculture With Autologous Patient‐Derived Xenograft (PDX) Tumor Cells

TILs were cultured with or without spermidine for 7 days in X‐VIVO 15 medium supplemented with IL‐2 (50 U/mL), IL‐7 (10 ng/mL), and IL‐15 (10 ng/mL). Oligomycin (2 μM; Sigma–Aldrich, USA) was added on Day 3 and maintained for the remaining 5 days of culture.

PDX tumors from NOD‐SCID mice were thawed from liquid nitrogen, centrifuged, and the resulting pellets were resuspended in X‐VIVO 15 medium. Tumors were then mechanically and enzymatically dissociated using the Tumor Dissociation Kit (Miltenyi Biotec, USA) in a gentleMACS Octo Dissociator (Miltenyi Biotec, USA) for 30 min. The dissociated cells were subsequently centrifuged and resuspended in fresh medium for downstream applications. Then the TILs and tumor cells were cocultured in the 96‐well plate in the 1:1 of effector cells:target cells (E:T) for 24 h, then, cells were collected for the flow cytometric analysis of degranulation (CD107a) by the protocol described above, and simultaneously the supernatants were collected for the IFN‐γ detection. Cell culture supernatants were centrifuged at 300 × *g* for 5 min to remove debris. The concentration of human IFN‐γ in the supernatants was measured using a Human IFN‐γ ELISA Kit (Thermo Fisher, USA) according to the manufacturer’s instructions. The reaction was stopped with 2 N sulfuric acid and absorbance was measured at 450 nm using a microplate reader. IFN‐γ concentrations were calculated by interpolation from a standard curve generated with known concentrations of recombinant human IFN‐γ provided in the kit.

### 2.7. In Vitro Cell Toxicity Assay

We used the CFSE–based cell cytotoxicity assay to measure the cytotoxic efficacy of the T cells. Dissociated tumor cells from PDX models were stained by 2 μM of CFSE for 30 min followed by the PBS washing and staining stop by FBS‐included medium for 10 min. The CFSE‐stained tumor cells were cocultured with T cells at the 5:1, 10:1, and 20:1 of E:T ratio for 6 h at 37°C incubator. After coculture, 1 μg/mL propidium iodide (PI; BD Biosciences, USA) was added to stain the target cells for 15 min at room temperature, and then, the samples were analyzed by Accuri C6 Flow Cytometer (BD Biosciences, USA).

### 2.8. Oxygen Consumption Rate (OCR) and Extracellular Acidification Rate (ECAR) Assays

The Seahorse XFe24 Analyzer (Agilent Technologies, USA) was employed to measure the OCR and ECAR of T cells. These cells, treated with spermidine or left untreated for 7 days, were seeded onto poly‐lysine‐coated XF24 plates to ensure adherence, with the plates pretreated for 24 h at room temperature.

During the mitochondrial stress test (MST), the OCR was recorded initially under baseline conditions, followed by the administration of sequential doses of oligomycin (1 mM), FCCP (1 mM carbonyl cyanide 4‐trifluoromethoxyphenylhydrazone), and a combination of rotenone and antimycin (0.5 mM each, R/A). These measurements facilitated the calculation of the basal respiration rate (baseline OCR minus R/A OCR), maximal respiration rate (FCCP OCR minus R/A OCR), and the cell’s oxidative capacity (maximal respiration minus basal respiration). In the glycolysis stress test (GST), the ECAR was assessed under baseline conditions to determine the basal glycolytic rate, adhering to the manufacturer’s protocol. Seahorse Wave software version 2.6 (Agilent Technologies, USA) processed and analyzed all obtained data.

### 2.9. Energy Metabonomic Analysis

Cells, both spermidine‐treated and untreated, were concentrated by centrifugation and rinsed once with PBS. For each analysis, 5 × 10^6^ cells were processed, with metabolites extracted using 80% methanol that had been chilled to −80°C at least an hour prior. Samples underwent three cycles of flash freezing in liquid nitrogen for 5 min and thawing on ice for 5 min, followed by centrifugation at 12,000 rpm for 20 min at 4°C. The supernatants were then collected and evaporated in preparation for liquid chromatography and tandem mass spectrometry (LC‐MS/MS) performed at Metware Bioscience Company (Wuhan, China).

Data collection utilized ultra performance LC (UPLC; Shimpack, UFLC SHIMADZU CBM30A) and MS (QTRAP). The chromatography used a SeQuant ZIC‐pHILIC column (5 μm, 2.1mm × 100 mm) with mobile phase A consisting of 10 mM ammonium carbonate and 0.05% ammonium hydroxide and mobile phase B being 100% acetonitrile. The gradient was set from 5:95 (A:B *v*/*v*) at the start, changing to 50:50 (*v*/*v*) at 9.5 min, and returning to 5:95 (*v*/*v*) by 11.1 min, maintained until 14.0 min. The flow rate was 0.4 mL/min, and the column was kept at 40°C, with a sample injection volume of 2 µL.

Mass spectrometry parameters included electrospray ionization (ESI) at 450°C, with ionization voltages set at 5500 V (positive) and −4500 V (negative), ion gas pressures at 40 psi (GS I) and 55 psi (GS II), and a curtain gas pressure of 35 psi. The collision‐activated dissociation (CAD) setting was medium. Ion pairs were scanned based on optimized declustering potential (DP) and collision energy (CE). Analysis of MS data was conducted using Analyst 1.6.3 software, with all data normalized to the proportion of lymphocytes and living cells in each sample.

### 2.10. Statistical Analysis

Statistical evaluations were conducted using Stata 11.0 for Windows (StataCorp LP), employing two‐tailed unpaired or paired Student’s *t*‐tests, one‐way and two‐way ANOVA for analysis. For one‐way and two‐way ANOVA, multiple comparisons were controlled by adjusting the false discovery rate (FDR) using the two‐stage setup method of Benjamini, Krieger, and Yekutieli. A *p*‐value of less than 0.05 was deemed statistically significant. Error bars depict the mean ± standard error of the mean (SEM), and significance levels were denoted as  ^∗^ *p*  < 0.05,  ^∗∗^ *p*  < 0.01,  ^∗∗∗^ *p*  < 0.001.

## 3. Results

### 3.1. Spermidine Promotes Mitochondrial Biosynthesis and Mitochondrial OXPHOS

Building on initial discovery that spermidine enhances autophagy flux in TILs, we extended our research to examine the connection between spermidine‐induced autophagy flux and the metabolic profile of these cells. Our investigation aimed to determine how spermidine influences the metabolic dynamics of TILs.

Initially, we assessed the impact of spermidine on glucose absorption in TILs, employed by flow cytometry. The results indicated a marked increase in glucose uptake in spermidine‐exposed TILs, observable across both CD4+ and CD8+ tumor‐infiltrating T cells subsets (Figure 1A,B and Supporting Information 1: Figure S1A,B). Our inquiry further extended to whether spermidine could boost mitochondrial OXPHOS or glycolysis by rejuvenating autophagy flux. By measuring OCR and ECAR, indicators of OXPHOS and glycolysis, respectively, we noted that spermidine exposure elevated basal OCR and maximal respiratory capability post mitochondrial membrane decoupling with FCCP (Figure 1C–E), whereas ECAR levels remained unchanged (Figure 1F), suggesting spermidine primarily enhances OXPHOS.

Figure 1Spermidine improves the mitochondria biogenesis and mitochondrial oxidative phosphorylation in TILs (A, B). Representative flow cytometric plots showed the proportions of 2‐NBDG + TILs with or without spermidine treatment (A). Statistical summary regarding the proportions of 2‐NBDG + TILs with or without spermidine treatment was shown (*n* = 7). Accumulated data are shown as mean ± SEM and a paired *T* test ( ^∗∗^ *p*  < 0.01) was conducted (B). (C–E) Representative O_2_ consumption rates (OCR) in TILs with or without spermidine treatment measured in real time under basal conditions in response to the mitochondrial inhibitors oligomycin, FCCP [carbonyl cyanide 4‐(trifluoromethoxy)phenylhydrazone], and R/A (rotenone/antimycin) was shown (*n* = 5). Accumulated data at each time point are shown as mean ± SEM and a paired *T* test ( ^∗∗^ *p*  < 0.01,  ^∗∗∗^ *p*  < 0.001) was conducted (C). The statistical summary of basal OCR in TILs with or without spermidine treatment was shown (*n* = 5).The collected data are presented as the mean ± SEM, and a paired *T*‐test was performed ( ^∗^ *p*  < 0.05) (D). The statistical summary of maximal OCR in TILs with or without spermidine treatment was shown (*n* = 5). Accumulated data are presented as the mean ± SEM, and a paired *T*‐test ( ^∗∗^ *p*  < 0.01) was performed (E). (F) The statistical summary of basal extracellular acid rate (ECAR) in the TILs with or without spermidine treatment was shown (*n* = 5). The data are summarized as the mean ± SEM, and a paired *T*‐test was conducted (G). The statistical summary of qPCR results of mitochondrial DNA (mtDNA) in TILs with or without spermidine treatment was shown (*n* = 5). The data are summarized as the mean ± SEM, and a paired *T*‐test ( ^∗^ *p*  < 0.05) was conducted (H, I). The representative flow cytometric plots of TMRM stained TILs with or without spermidine treatment was shown (H). Statistical summary of the MFI of TMRM stained TILs with or without spermidine treatment was shown (*n* = 8). The data are summarized as the mean ± SEM, and a paired *T*‐test ( ^∗∗^ *p*  < 0.01) was conducted (I). (J, K) The representative flow cytometric plots of MitoTracker Green (MTG) stained TILs with or without spermidine treatment was shown. (J) Statistical summary of the MFI of MTG stained TILs with or without spermidine treatment was shown (*n* = 6). The data are summarized as the mean ± SEM, and a paired *T*‐test ( ^∗∗^ *p*  < 0.01) was conducted (K). (L, M) Representative flow cytometric plots of MitoTracker Orange CMTMRos (MTO)‐stained TILs, with or without spermidine treatment, were shown (L). Statistical summary of the MFI of MTO stained TILs with or without spermidine treatment was shown (*n* = 6). The data are summarized as the mean ± SEM, and a paired *T*‐test ( ^∗∗∗^ *p*  < 0.001) was conducted (M). (N) Statistical summary of differentially expressed metabolites implicated in tricarboxylic acid cycle (TCA) and energy metabolism in TILs with or without spermidine treatment was shown according to liquid chromatography and tandem mass spectrometry (LC‐MS/MS) analysis. Samples are normalized to TILs without spermidine treatment. The data are summarized as the mean ± SEM, and a paired *T*‐test ( ^∗^ *p*  < 0.05,  ^∗∗^ *p*  < 0.01) was conducted (*n* = 4).

**Figure:**
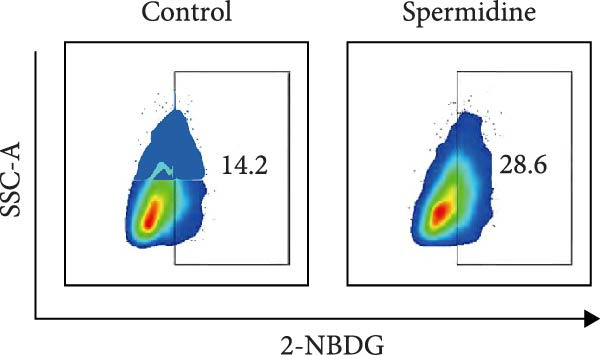
(A)

**Figure:**
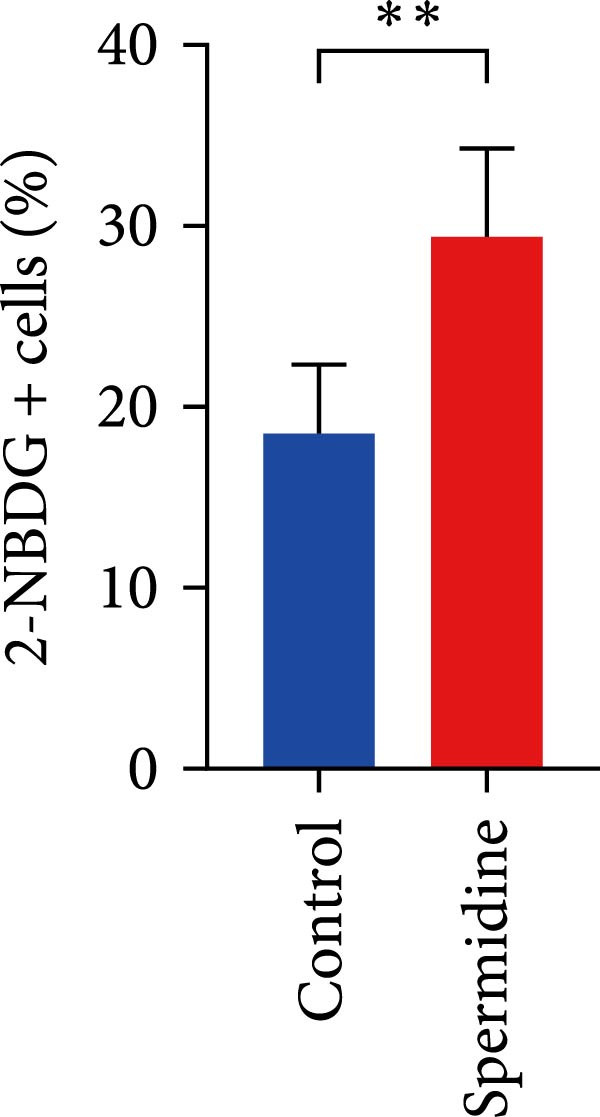
(B)

**Figure:**
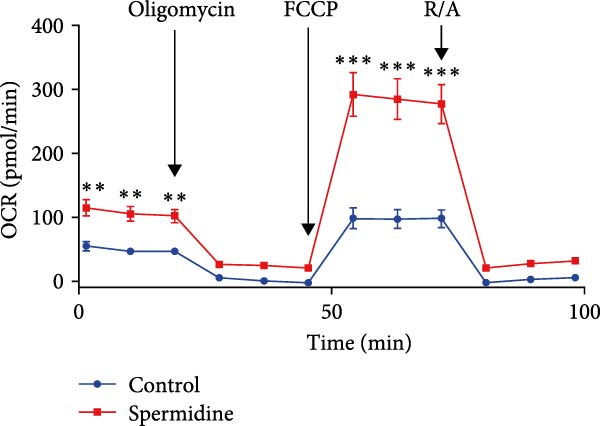
(C)

**Figure:**
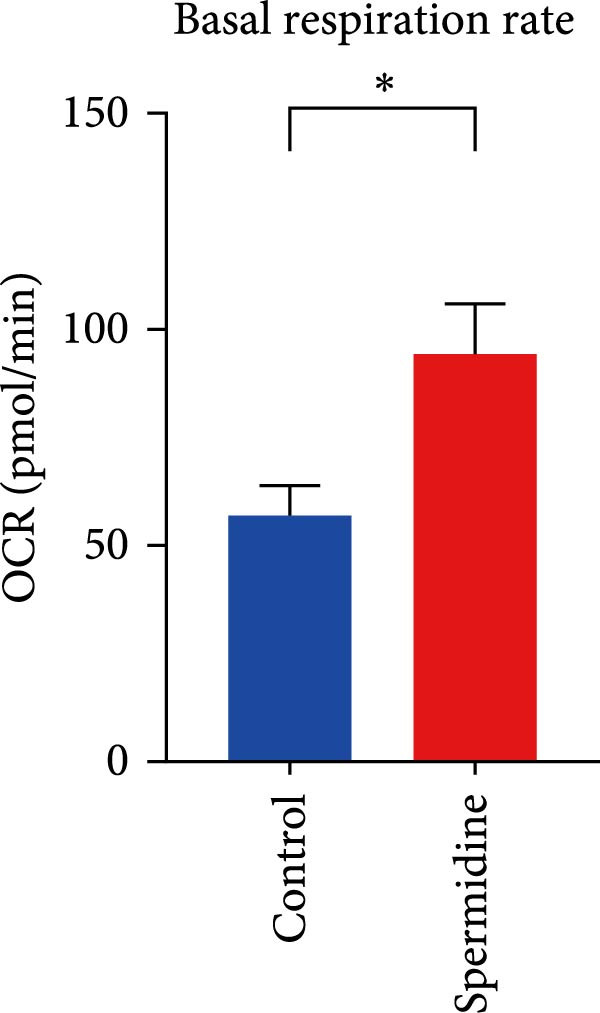
(D)

**Figure:**
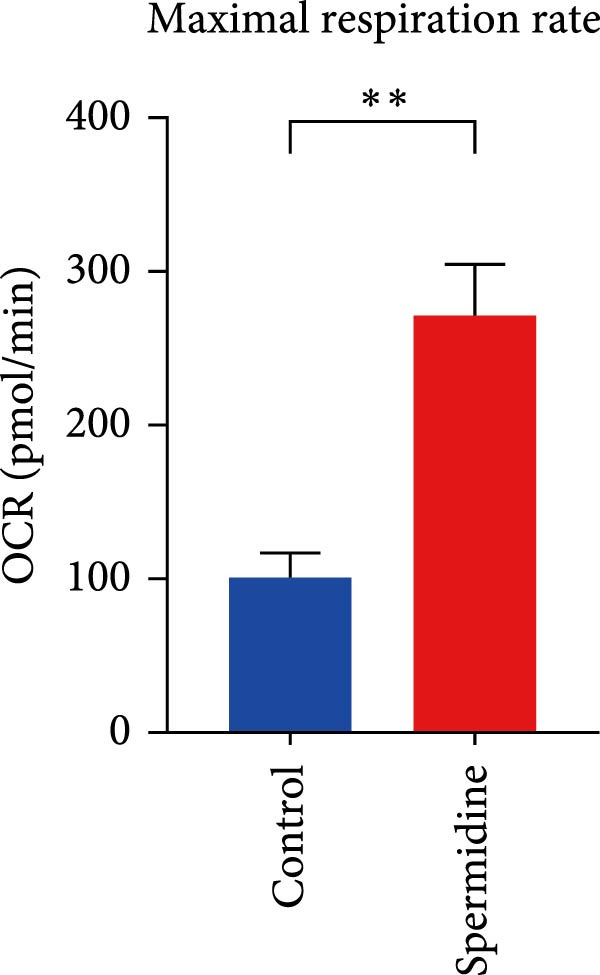
(E)

**Figure:**
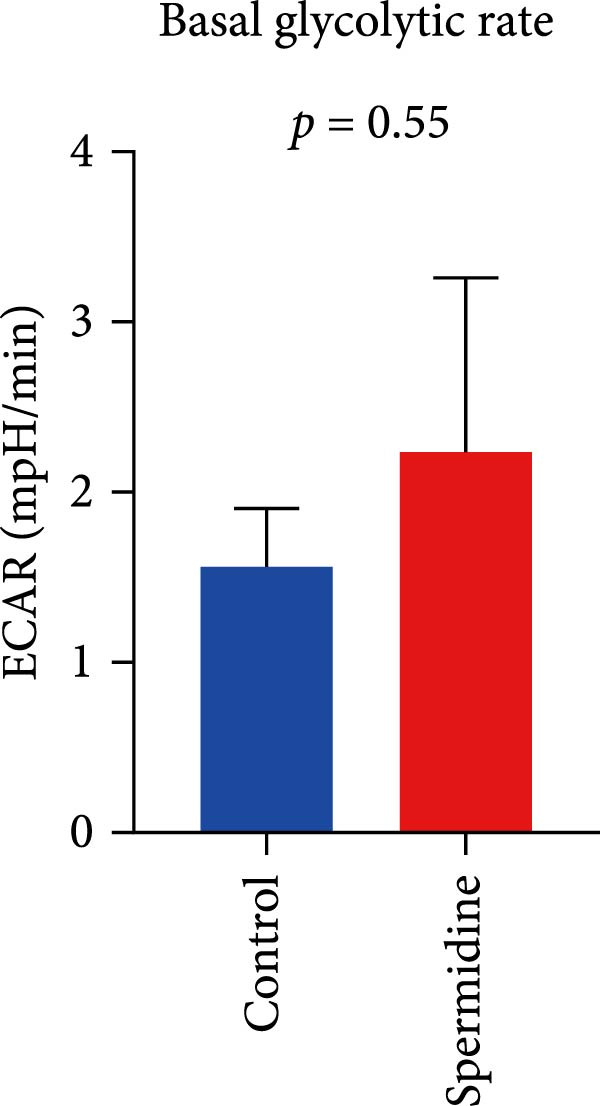
(F)

**Figure:**
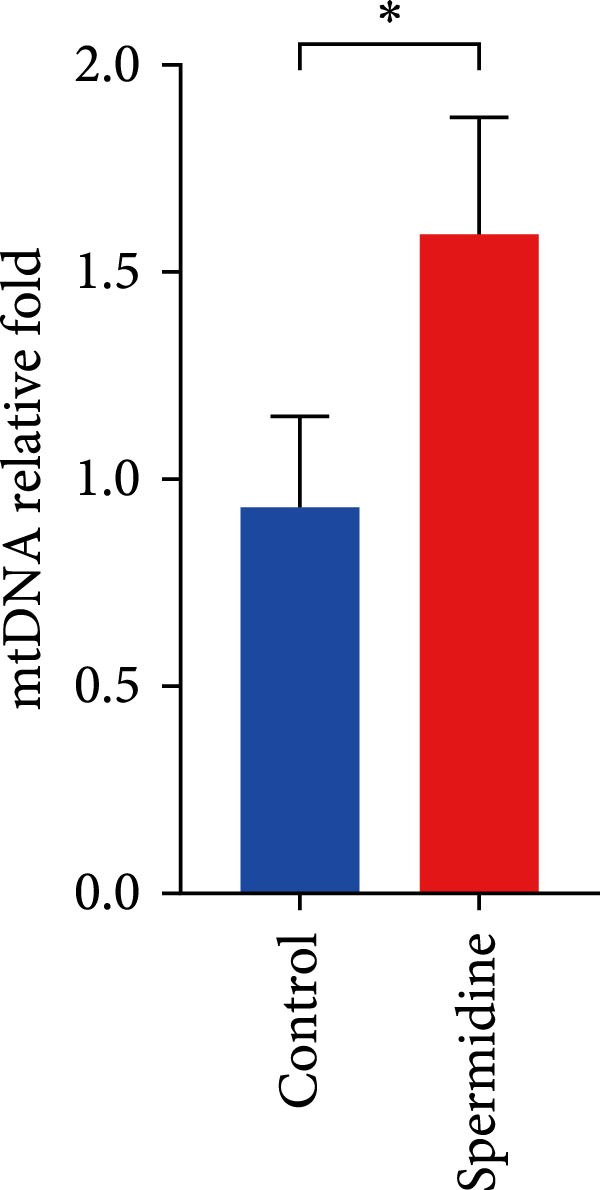
(G)

**Figure:**
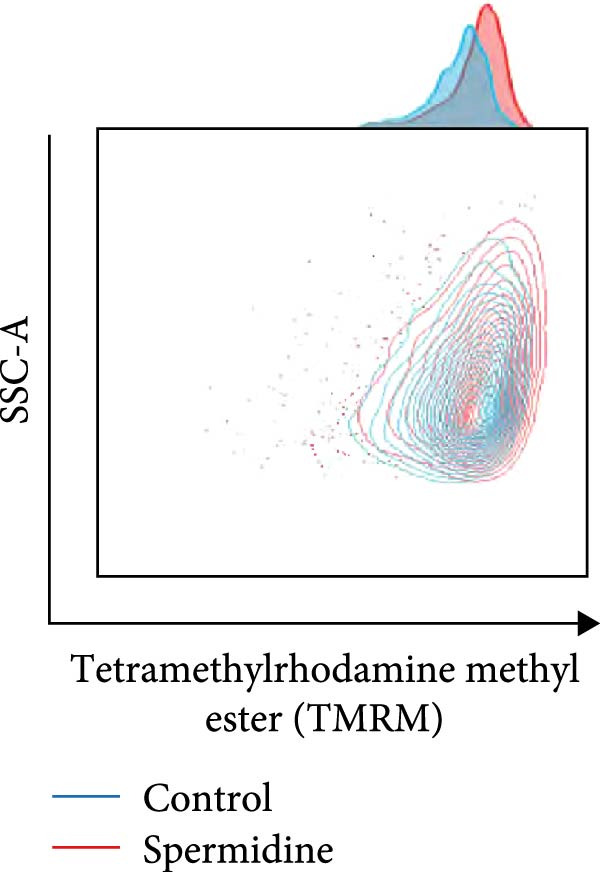
(H)

**Figure:**
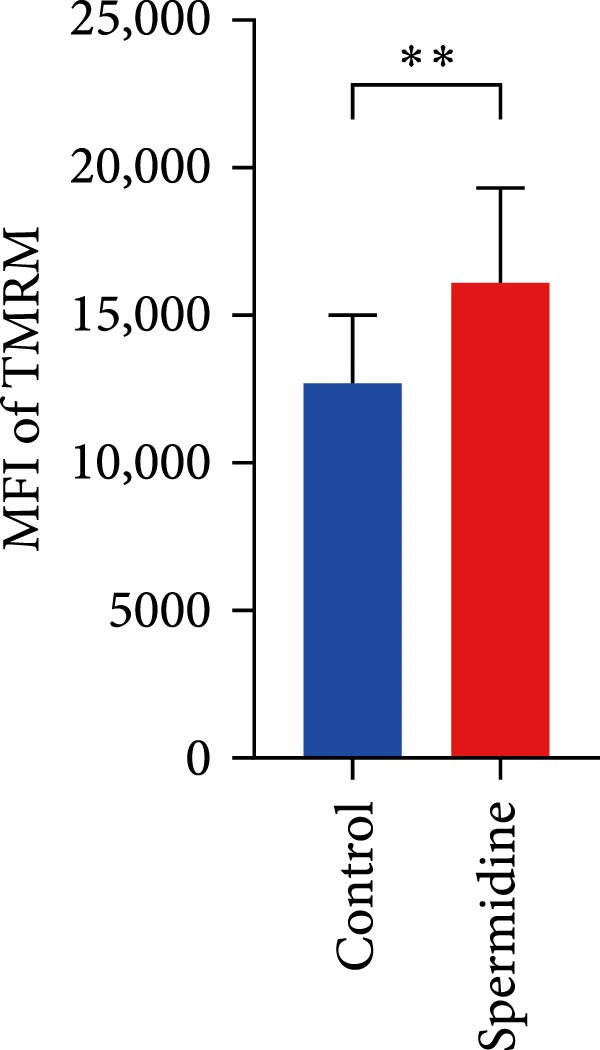
(I)

**Figure:**
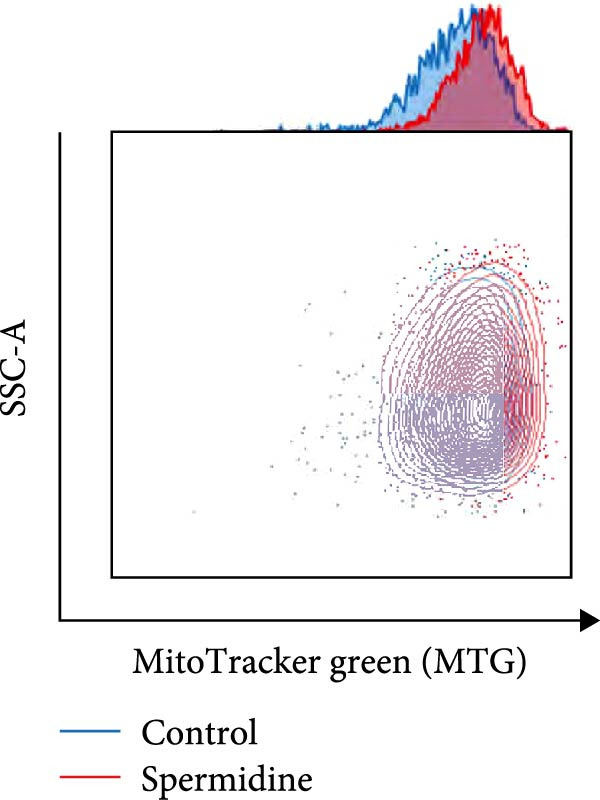
(J)

**Figure:**
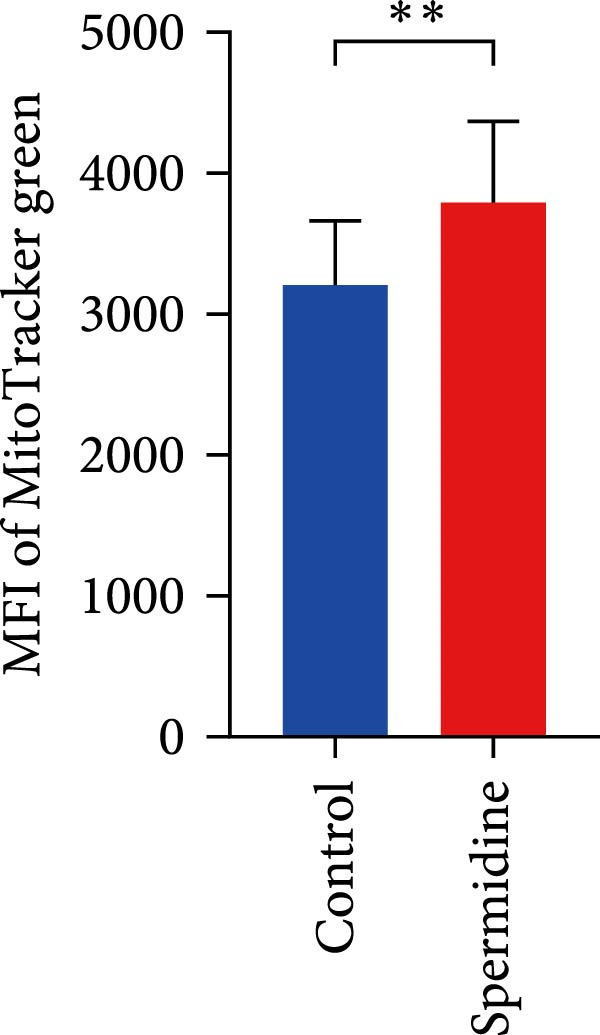
(K)

**Figure:**
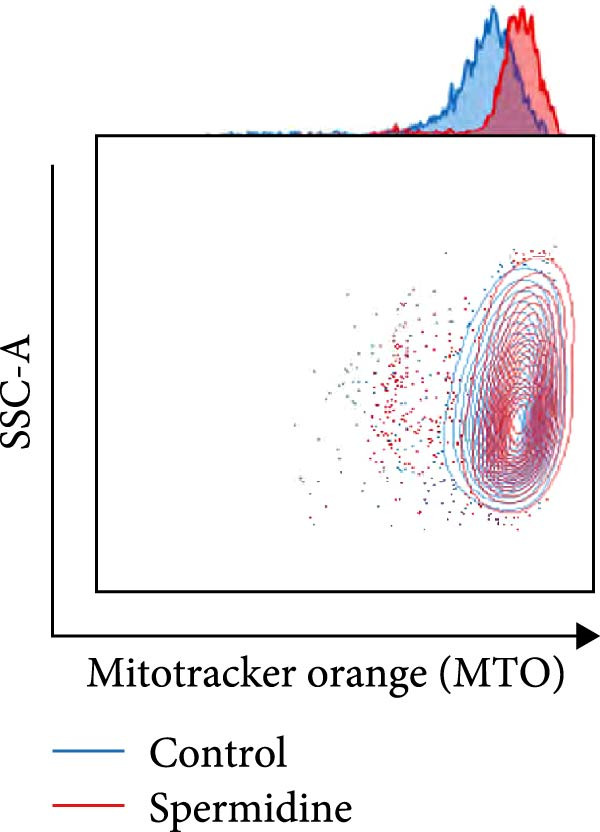
(L)

**Figure:**
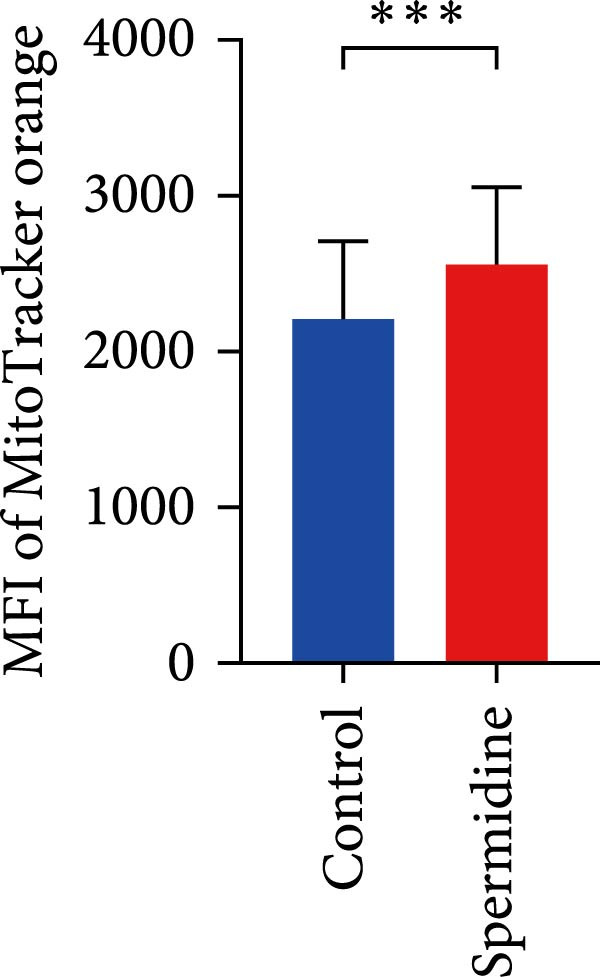
(M)

**Figure:**
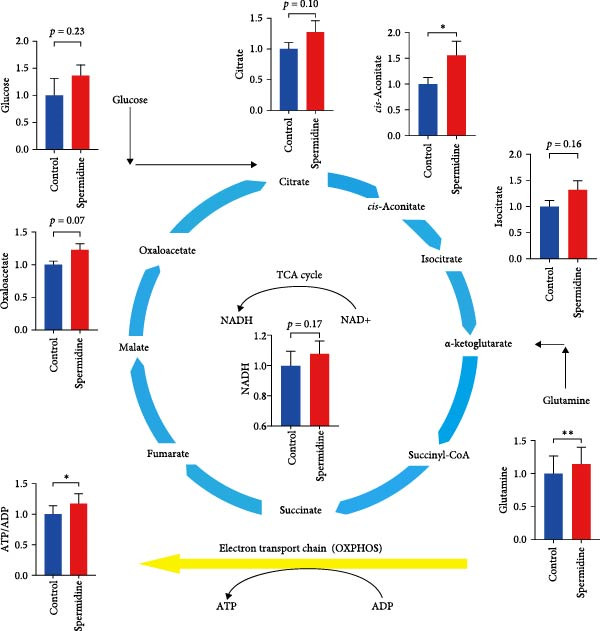
(N)

Considering the role of mitochondrial density in OXPHOS, we examined whether the observed increases in basal OCR and maximal respiratory capacity under spermidine treatment corresponded with augmented mitochondrial biosynthesis. qPCR revealed significantly higher mitochondrial DNA levels in spermidine‐treated TILs (Figure 1G).

To assess the effects of spermidine on MMP, we initially used TMRM staining and observed that spermidine treatment significantly increased the MMP of TILs (Figure 1H,I). To strengthen this observation, we further employed CCCP, a mitochondrial uncoupler, to determine the minimal MMP baseline and oligomycin, an ATP synthase inhibitor, to assess the maximal achievable MMP. CCCP treatment reduced TMRM fluorescence to a minimal and indistinguishable level in both groups, confirming that the signal was MMP‐dependent. Oligomycin treatment resulted in a greater increase in TMRM fluorescence in spermidine‐treated cells, indicating that these cells were actively engaged in ATP production and maintained a higher MMP reserve compared to controls (Supporting Information 1: Figure S1C,D).

Additionally, a notable rise in mitochondrial mass, indicated by MitoTracker Green staining, was confirmed through flow cytometry (Figure 1J,K). In addition to TMRM probe detection, our analysis using a combination of potential‐dependent and independent dyes showed a higher proportion of spermidine‐treated TILs had polarized mitochondria (Figure 1L,M), a finding consistent across CD8+ and CD4+ T cells (Supporting Information 1: Figure S1E–H). These observations underline that spermidine treatment results in a higher density of both total and polarized mitochondria compared to untreated TILs.

Building on the findings of enhanced OXPHOS and mitochondrial biogenesis in spermidine‐treated TILs, we explored the impact on metabolites. Through LC and mass spectrometry, we discovered elevated levels of metabolic intermediates linked to the tricarboxylic acid (TCA) cycle in spermidine‐treated TILs, an essential pathway for fueling OXPHOS (Figure 1N), alongside comparable levels of glycolytic intermediates to untreated TILs (Supporting Information 1: Figure S2 and Supporting Information 2: Table S1). This metabolic enhancement was further evidenced by a higher ATP/ADP ratio in spermidine‐treated TILs (Figure 1N). The gating strategy regarding the abovementioned results for identifying CD3+ T cells, as well as CD4+ and CD8+ T cells, is illustrated (Supporting Information 1: Figure S1I).

In conclusion, our results reveal that spermidine treatment significantly bolsters OXPHOS metabolism of TILs, reflected through an increase in both total and polarized mitochondrial mass and accumulation of TCA cycle metabolites. This metabolic enhancement may underpin the recuperation from dysfunction and exhaustion, thereby amplifying the therapeutic efficacy of TILs.

### 3.2. Suppressing OXPHOS Negates the Ability of Spermidine to Mitigate Exhaustion in TILs

Building on our previous studies [23, 24], we have observed that spermidine reduces T cell exhaustion by downregulating markers such as PD1, TIM3, and LAG3, and enhances T cell function, including proliferation and IFN‐γ production, in TILs and T cells isolated from PBMCs of elderly patients. Additionally, as previously mentioned, we found that spermidine positively impacts mitochondrial function and metabolism. We hypothesize that these effects on mitochondrial function may play a key role in mitigating T cell exhaustion and enhancing T cell function.

To assess the criticality of mitochondrial ATP, produced through OXPHOS, in revitalizing the functionality of TILs via spermidine‐induced autophagy enhancement, we cultured spermidine‐treated TILs with the ATP synthase inhibitor oligomycin, which could also be regarded as an inhibitor of mitochondrial function. We then evaluated the impact on T cell functionality. Our observations revealed that while spermidine treatment led to a reduction in the expression of inhibitory receptors PD1, TIM3, and LAG3, as well as a significant decrease in the frequency of TILs co‐expressing these inhibitory receptors, the introduction of oligomycin markedly increased the expression of these markers in spermidine‐treated TILs (Figure 2A–C). Moreover, oligomycin was found to significantly impede the proliferative capacity (Figure 2D,E) and IFN‐γ production in spermidine‐treated TILs under the PMA plus ionomycin stimulation and OKT3 stimulation (Figure 2F–I), with similar trends observed across both CD8+ and CD4+ T cell populations (Supporting Information 1: Figure S3A–H).

Figure 2Oligomycin compromises the spermidine‐induced reversal of dysfunction and exhaustion of TILs. (A–C) Flow cytometric plots illustrating TILs expressing inhibitory immunoreceptors (PD1, TIM3, and LAG3) are shown for the three groups: control, spermidine, and spermidine + oligomycin. (A) Statistical summary of double positive TILs (PD1 + TIM3; PD1 + LAG3; TIM3 + LAG3) in three groups of control, spermidine, and spermidine + oligomycin was shown (*n* = 7). A two‐way ANOVA ( ^∗^ *p*  < 0.05) was conducted, we corrected the multiple comparisons by controlling the false discovery rate (FDR). Two‐stage set‐up method of Benjamini, Krieger, and Yekutieli was conducted. (B) Statistical summary of proportions of PD1+, TIM3+, or LAG3+ TILs in three groups of control, spermidine, and spermidine + oligomycin was shown (*n* = 5). A two‐way ANOVA ( ^∗^ *p*  < 0.05) was conducted, we controlled for multiple comparisons by adjusting the false discovery rate (FDR) using the two‐stage setup method of Benjamini, Krieger, and Yekutieli (C). (D, E) Representative flow cytometric plots showed proportions of Ki67+ TILs in three groups of control, spermidine, and spermidine+oligomycin. (D) Statistical summary of proportions of Ki67+ TILs in three groups of control, spermidine, and spermidine + oligomycin was shown (*n* = 5). A paired one‐way ANOVA ( ^∗^ *p*  < 0.05) was performed, with multiple comparisons controlled by adjusting the false discovery rate (FDR) using the two‐stage setup method of Benjamini, Krieger, and Yekutieli (E). (F, G), Representative flow cytometric plots showed proportions of IFN‐γ+ TILs in three groups of control, spermidine, and spermidine + oligomycin under PMA + ionomycin stimulation. (F) The proportions of IFN‐γ+ TILs in three groups of control, spermidine, and spermidine + oligomycin under PMA + ionomycin stimulation was summarized (*n* = 3). An unpaired *T*‐test was performed between each pair of groups (G). (H, I) Representative flow cytometric plots demonstrated proportions of IFN‐γ+ TILs in three groups of control, spermidine and spermidine + oligomycin under OKT3 stimulation. (H) The proportions of IFN‐γ+ TILs in three groups of control, spermidine, and spermidine + oligomycin under OKT3 stimulation was summarized (*n* = 5). A paired one‐way ANOVA ( ^∗^ *p*  < 0.05) was performed, with multiple comparisons controlled by adjusting the false discovery rate (FDR) using the two‐stage setup method of Benjamini, Krieger, and Yekutieli (I). (J) The IFN‐γ production in three groups of control, spermidine, and spermidine + oligomycin upon coculture with autologous PDX tumors was summarized (*n* = 4). A paired one‐way ANOVA ( ^∗∗^ *p*  < 0.01,  ^∗∗∗^p < 0.001) was performed, with multiple comparisons controlled by adjusting the false discovery rate (FDR) using the two‐stage setup method of Benjamini, Krieger, and Yekutieli. (K) The proportions of CD107a+ TILs in three groups of control, spermidine, and spermidine + oligomycin upon coculture with autologous PDX tumors was summarized (*n* = 4). A paired one‐way ANOVA ( ^∗^ *p*  < 0.05,  ^∗∗^ *p*  < 0.01) was performed, with multiple comparisons controlled by adjusting the false discovery rate (FDR) using the two‐stage setup method of Benjamini, Krieger, and Yekutieli. (L) Killing assays were performed by coculturing TILs with autologous PDX tumor cells at varying effector‐to‐target (E:T) ratios across three conditions: control, spermidine, and spermidine + oligomycin (*n* = 4). Tumor cell death was quantified in relation to control group without coculture with TILs, and statistical analysis was conducted using a paired two‐way ANOVA. Multiple comparisons were corrected using Tukey’s post hoc test ( ^∗^ *p*  < 0.05,  ^∗∗∗^ *p*  < 0.001,  ^∗∗∗∗^ *p*  < 0.0001).

**Figure:**
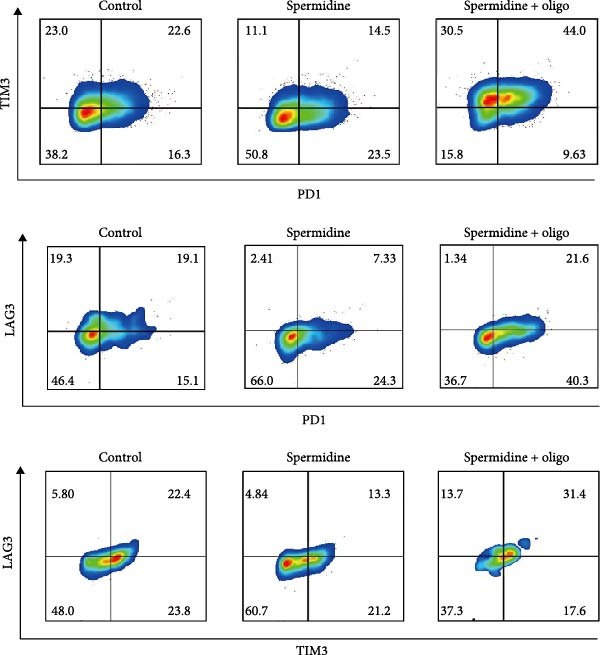
(A)

**Figure:**
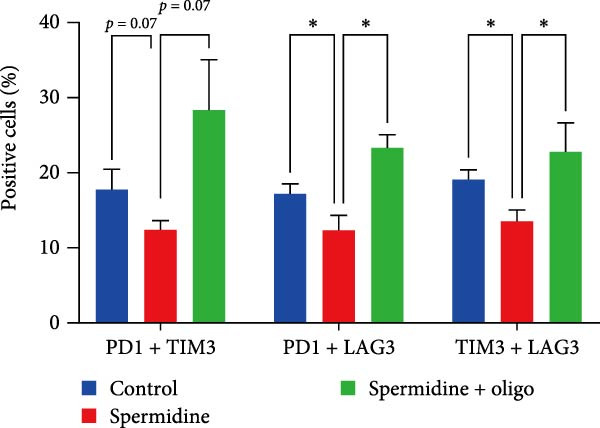
(B)

**Figure:**
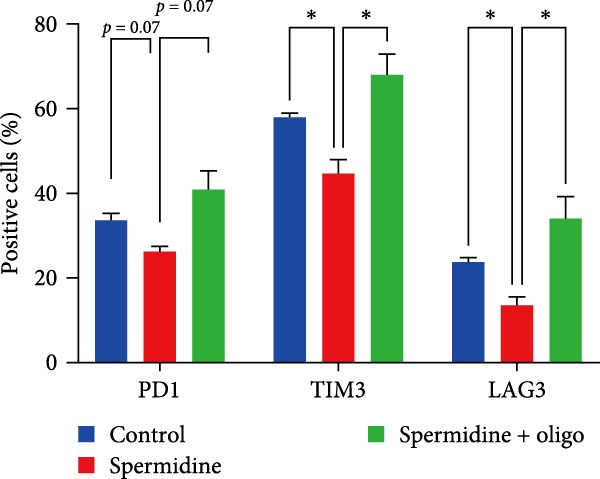
(C)

**Figure:**
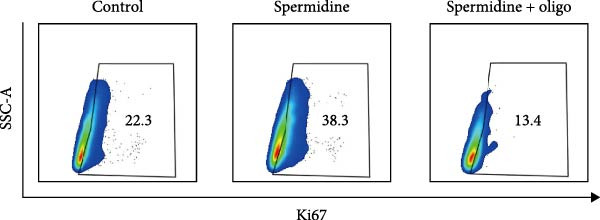
(D)

**Figure:**
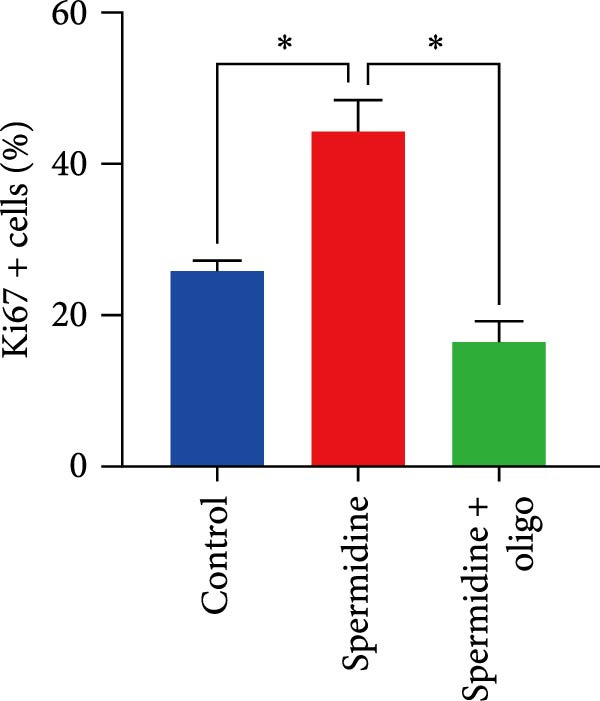
(E)

**Figure:**
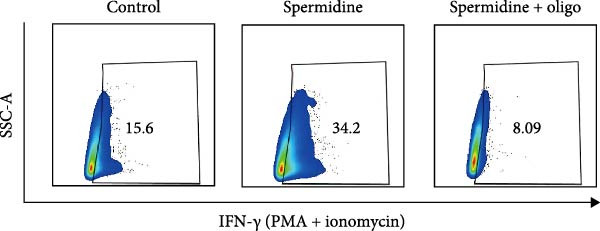
(F)

**Figure:**
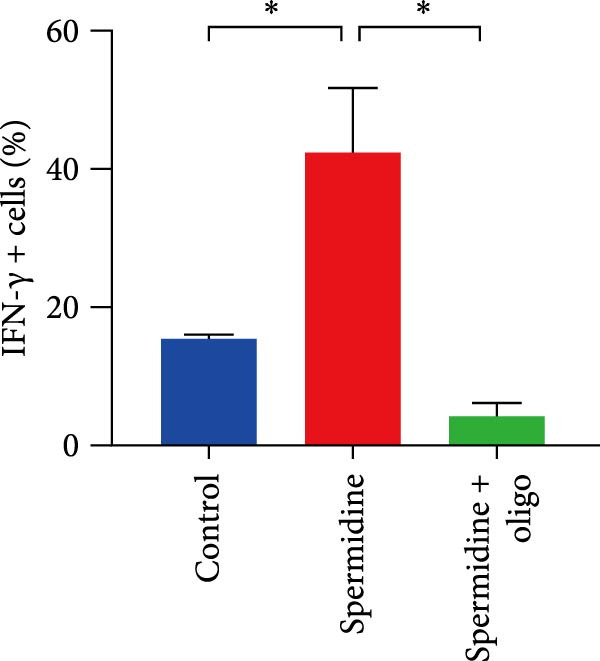
(G)

**Figure:**
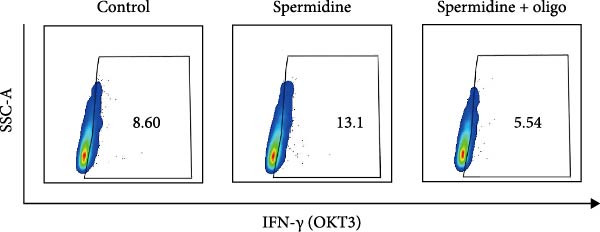
(H)

**Figure:**
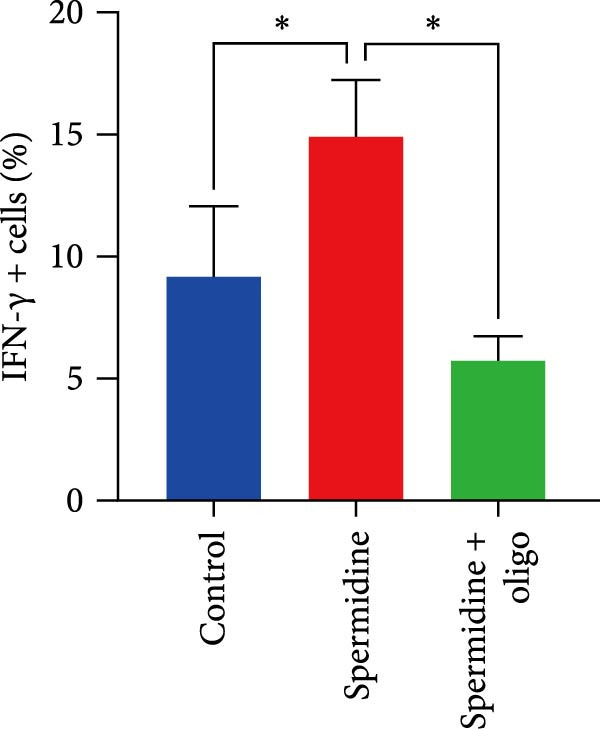
(I)

**Figure:**
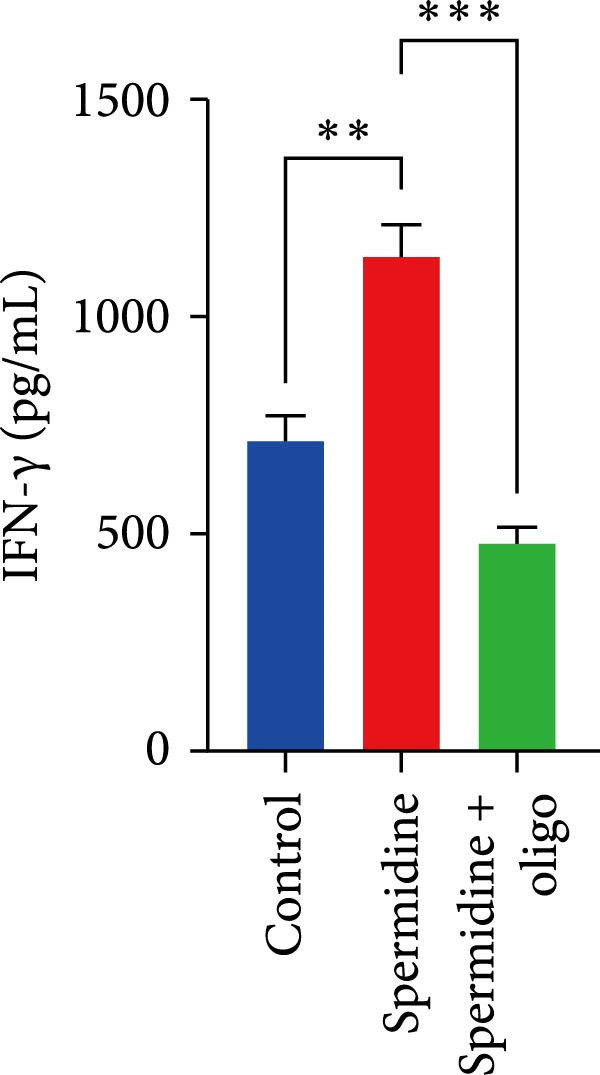
(J)

**Figure:**
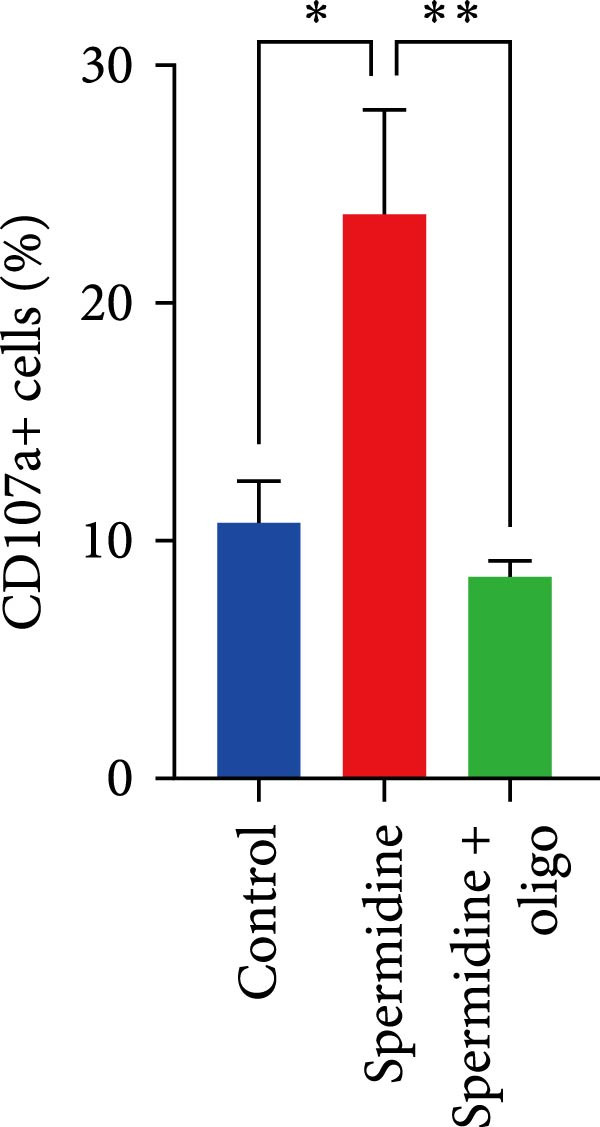
(K)

**Figure:**
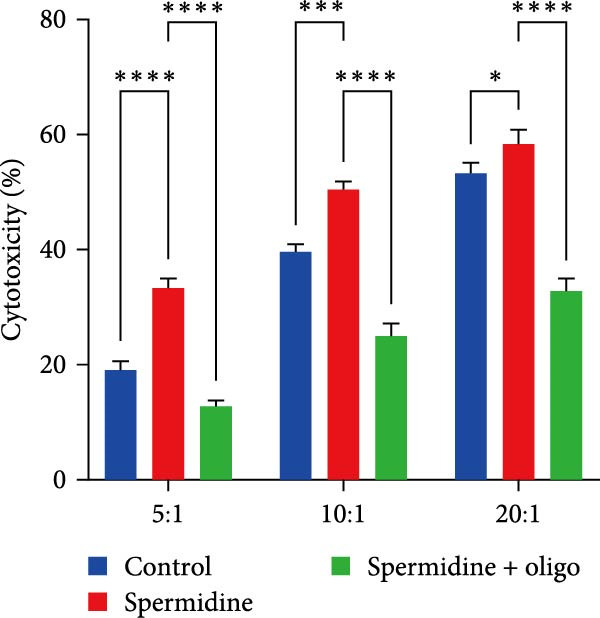
(L)

In addition to the in vitro stimulation model, we further performed functional assays by coculturing TILs with autologous PDX tumor cells. Consistent with previous findings, oligomycin treatment impaired IFN‐γ production, degranulation, and cytotoxic activity against autologous targets, indicating that mitochondrial function is essential for the effector functions of TILs (Figure 2J–L).

These results unequivocally establish that mitochondrial ATP generation through OXPHOS is pivotal for the restoration of function and reversal of exhaustion in TILs treated with spermidine.

## 4. Discussion

T cell metabolism is closely linked to exhaustion, and our findings highlight the role of spermidine in enhancing mitochondrial biogenesis and function within TILs, offering a novel strategy for reversing exhaustion. Enhancing autophagy flux mitigates TIL dysfunction and exhaustion, improving antitumor capabilities, yet the precise regulatory mechanisms require further exploration [10, 26]. Notably, reduced mitochondrial mass has been associated with increased expression of inhibitory receptors [27].

Spermidine promotes autophagy via multiple pathways, including HAT inhibition, mTOR suppression, EIF5A hypusination, and activation of TFEB, FOXO3, and AMPK, all of which contribute to metabolic resilience [28]. Our metabolomic analysis revealed that spermidine‐treated TILs exhibited enhanced OXPHOS metabolism, increased mitochondrial mass, and TCA cycle metabolite accumulation. Inhibition of ATP synthase led to elevated PD1, TIM3, and LAG3 expression while reducing TIL proliferation and IFN‐γ production, reinforcing the role of metabolic reprograming in restoring TIL function [29].

Mitochondrial dysfunction is a hallmark of exhausted T cells, characterized by decreased mitochondrial mass, altered dynamics, and elevated ROS, which impair cytokine production and cytotoxic activity [30]. By supporting mitochondrial integrity and reducing ROS accumulation, spermidine may enhance TIL persistence and function in the tumor microenvironment. Additionally, autophagy plays a key role in maintaining metabolic fitness under hypoxia and nutrient deprivation by removing dysfunctional mitochondria, sustaining ATP production, and limiting oxidative damage [20].

TCA cycle metabolites are crucial regulators of T cell function. Spermidine‐induced metabolic shifts impact key intermediates such as citrate, α‐ketoglutarate, and succinate, which influence differentiation, proliferation, and cytokine production. For example, cis‐aconitate‐derived itaconate inhibits succinate dehydrogenase and reduces oxidative stress, modulating T cell activation [31]. Glutamine, essential for energy metabolism and nucleotide synthesis, balances effector and regulatory T cell responses [32]. Similarly, oxaloacetate supports ATP production and biosynthesis, enhancing T cell activation s [33].

Beyond mechanistic insights, our findings advocate for metabolic therapies as adjuncts to conventional cancer treatments. By leveraging metabolic modulators such as spermidine, adoptive cell therapies may achieve improved efficacy [34, 35].

However, clinical translation requires optimization of dosage, delivery methods, and safety assessments. While dietary spermidine is generally safe, high doses could lead to immunosuppression or metabolic disturbances, necessitating long‐term studies [20, 36, 37].

In conclusion, spermidine‐mediated metabolic reprograming presents a promising strategy to counteract T cell exhaustion, reinforcing the broader role of metabolism in immune regulation. Further investigation into metabolic pathways will refine strategies to enhance TIL–based immunotherapies, with spermidine emerging as a key candidate in this therapeutic frontier [38].

## Ethics Statement

The studies involving human participants were reviewed and approved by the Institutional Review Board of Beijing Cancer Hospital, China. Written informed consent to participate in this study was provided by the patient/participants’ OR patient/participants legal guardian/next of kin.

## Conflicts of Interest

The authors declare no conflicts of interest.

## Author Contributions

Conceptualization: Yizhe Sun and Chaoting Zhang. Data curation: Hao Fu, Xinyu Li, and Meng Wan. Formal analysis: Yizhe Sun and Hao Fu. Funding acquisition, supervision: Chaoting Zhang. Investigation: Yizhe Sun and Xinyu Li. Methodology: Yizhe Sun, Hao Fu, Meng Wan, Hongchao Xiong, and Chaoting Zhang. Project administration: Hongchao Xiong and Chaoting Zhang. Resources: Hongchao Xiong. Validation: Yizhe Sun, Hao Fu, and Hongchao Xiong. Visualization: Xinyu Li. Writing – original draft: Yizhe Sun. Writing – review and editing: Chaoting Zhang, Hao Fu, and Meng Wan. Yizhe Sun, Hao Fu and Xinyu Li are co‐first authors.

## Funding

This study was funded by the Beijing Natural Science Foundation (Grant L248027), the Beijing Nova Program (Grant 20230484366), the Natural Science Foundation of China (Grant 82473320), Science Foundation of Peking University Cancer Hospital (Grant BJCH2024GG05), and the Wu Jieping Medical Foundation (Grant 320.6750.2024‐17‐46).

## Supporting Information

Additional supporting information can be found online in the Supporting Information section.

## Supporting information


**Supporting Information 1** Figure S1. Spermidine enhances glucose uptake and mitochondria biogenesis in CD8+ or CD4+ TILs. Figure S2. Spermidine does not affect the glycolytic pathway in TILs. Figure S3. Oligomycin compromises the spermidine‐induced reversal of dysfunction and exhaustion of CD8+ and CD4+ TILs.

**Supporting Information 2** Table S1. Data related to the metabolites according to the metabonomic analysis.

## Data Availability

The datasets generated and analyzed during the current study are available from the corresponding author upon reasonable request.
