# Supplementary material for: Spermidine Remodels the Mitochondrial Metabolism of Tumor‐Infiltrating Lymphocytes
Source: J Immunol Res. 2025 Oct 29;2025:7550012. doi: 10.1155/jimr/7550012 (PMC12571991; doi:10.1155/jimr/7550012)
Supplement: Supplementary file 1 — Supporting Information 1 Figure S1. Spermidine enhances glucose uptake and mitochondria biogenesis in CD8+ or CD4+ TILs. Figure S2. Spermidine does not affect the glycolytic pathway in TILs. Figure S3. Oligomycin compromises the spermidine‐induced reversal of dysfunction and exhaustion of CD8+ and CD4+ TILs. [file JIMR-2025-7550012-s002.docx]

**Supplementary Figures**


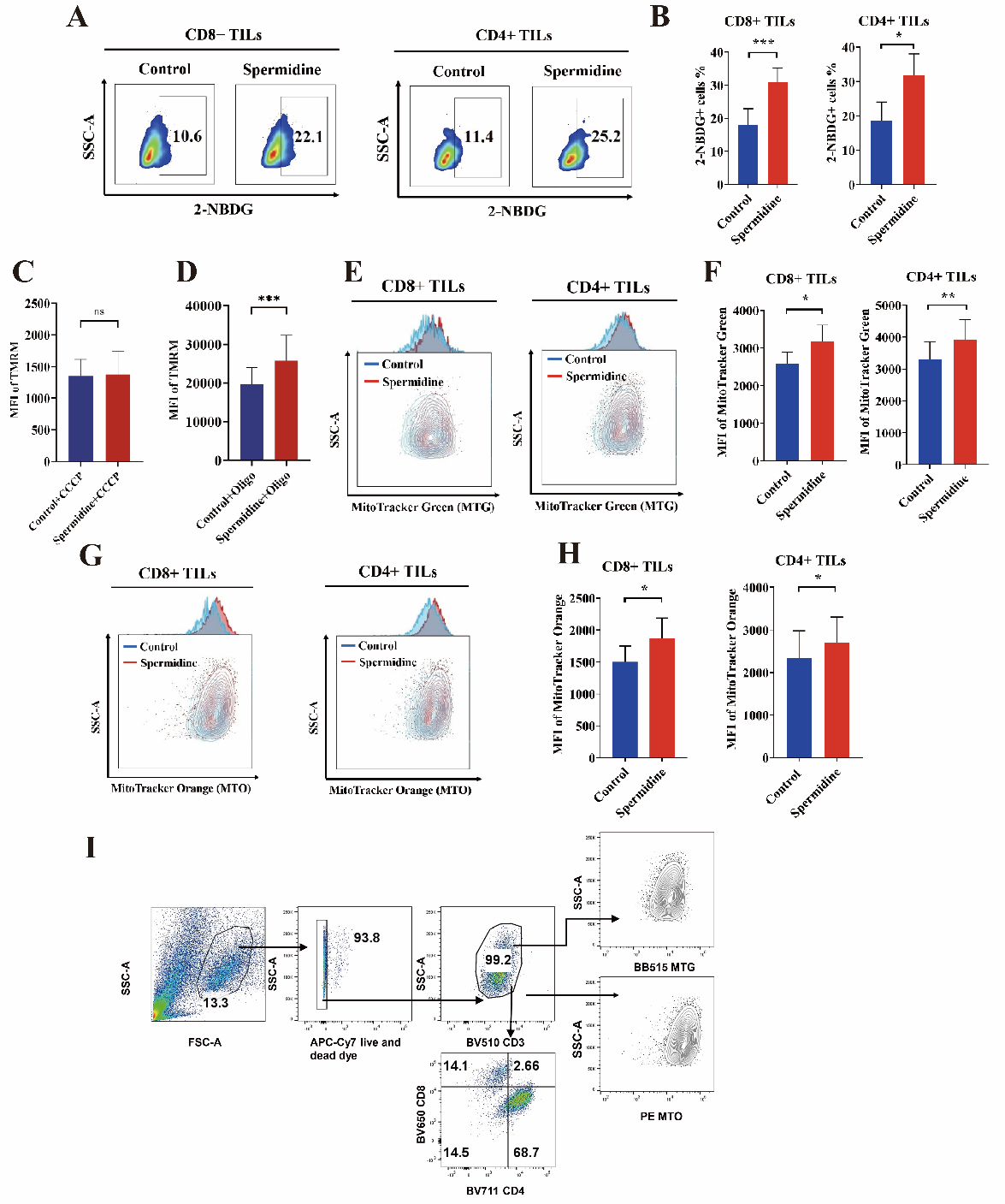


**Supplementary Figure 1.** **Spermidine enhances glucose uptake and mitochondria biogenesis in CD8+ or CD4+ TILs**

**A-B**, Representative flow cytometric plots showed the proportions of 2-NBDG+ TILs (CD8+ or CD4+) with or without spermidine treatment **(A)**. Statistical summary regarding the proportions of 2-NBDG+ TILs (CD8+ or CD4+) with or without spermidine treatment was shown (n=7). Accumulated data are presented as the mean ± SEM, and a paired T-test (*p<0.05, ***p < 0.001) was performed **(B)**.

**C-D,** Statistical summary of the MFI of TMRM stained TILs with or without spermidine treatment was shown (n=8) under CCCP **(C)** and oligomycin treatment **(D)**. Accumulated data are presented as the mean ± SEM, and a paired T-test (***p < 0.001) was performed

**E-F,** The representative flow cytometric plots of MitoTracker Green (MTG) stained TILs (CD8+ or CD4+) with or without spermidine treatment was shown **(E)**. Statistical summary of the MFI of MTG stained TILs (CD8+ or CD4+) with or without spermidine treatment was shown (n=6). Accumulated data are presented as the mean ± SEM, and a paired T-test (*p<0.05, **p < 0.01) was performed **(F)**.

**G-H,** The representative flow cytometric plots of MitoTracker Orange CMTMRos (MTO) stained TILs (CD8+ or CD4+) with or without spermidine treatment was shown **(G)**. Statistical summary of the MFI of MTO stained TILs (CD8+ or CD4+) with or without spermidine treatment was shown (n=6). Accumulated data are presented as the mean ± SEM, and a paired T-test (*p<0.05) was performed **(H)**.

**I,** The gating strategy used to identify CD3+ T cells, as well as CD4+ and CD8+ T cells is illustrated.


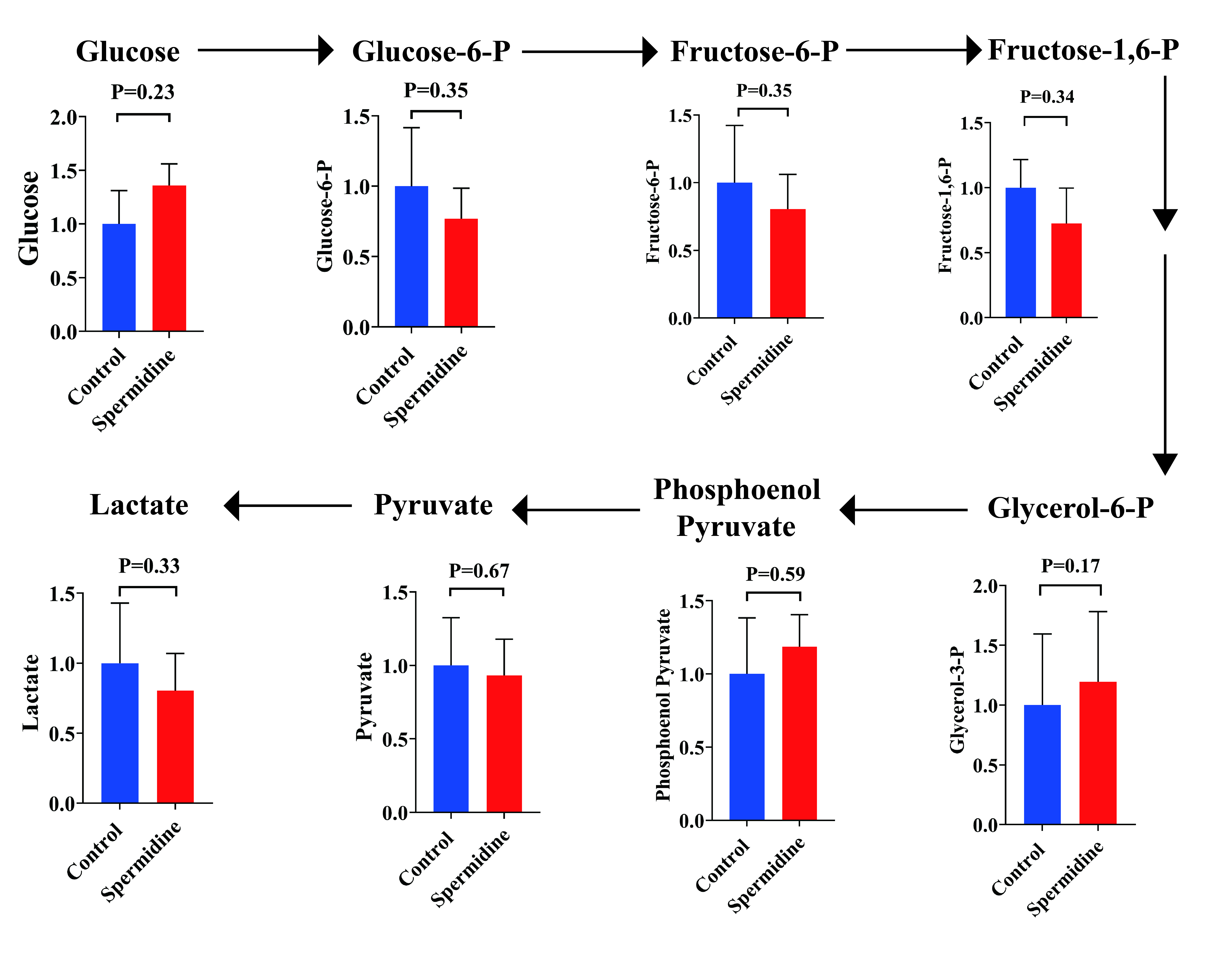


**Supplementary Figure 2.** **Spermidine did not affect the** **glycolytic pathway in TILs**

Statistical summary of metabolites implicated in glycolytic pathway in TILs with or without spermidine treatment was shown according to liquid chromatography and tandem mass spectrometry (LC-MS/MS) analysis. Samples are normalized to TILs without spermidine treatment. The data are summarized as the mean ± SEM, and a paired T-test was conducted (n=4).


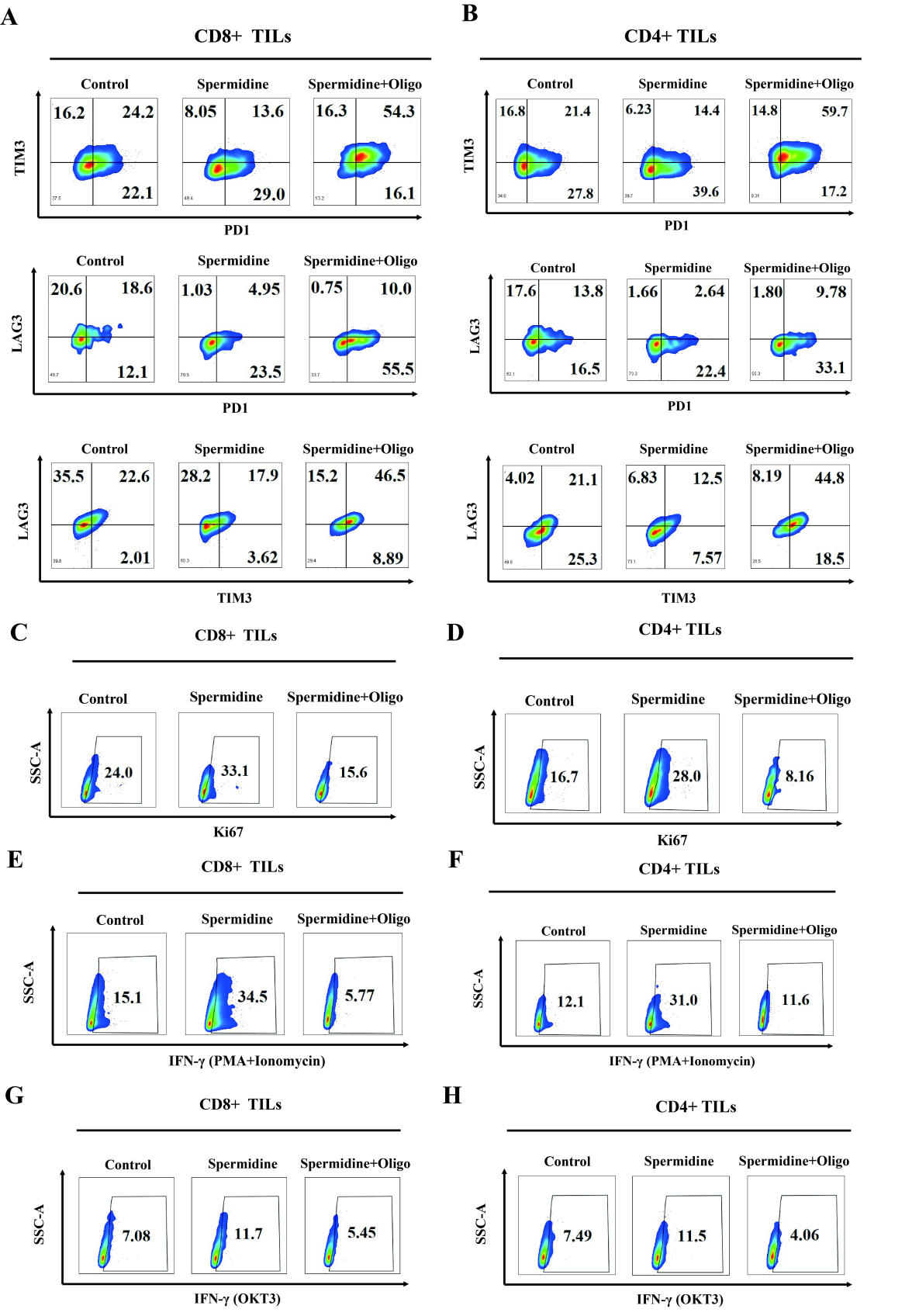


**Supplementary Figure 3. Oligomycin compromises the spermidine-induced reversal of dysfunction and exhaustion of CD8+ and CD4+ TILs.**

**A-B,** Representative flow cytometric plots of CD8+ TILs **(A)** and CD4+ TILs **(B)** expressing inhibitory immunoreceptors (PD1, TIM3, LAG3) in three groups of control, spermidine and spermidine+Oligomycin was shown.

**C-D,** Representative flow cytometric plots showed proportions of CD8+ Ki67+ TILs **(C)** and CD4+ Ki67+ TILs **(D)** in three groups of control, spermidine and spermidine+Oligomycin.

**E-F,** Representative flow cytometric plots showed proportions of CD8+ IFN-γ+ TILs **(E)**  and CD4+ IFN-γ+ TILs **(F)** in three groups of control, spermidine and spermidine+Oligomycin under PMA+ionomycin stimulation.

**G-H,** Representative flow cytometric plots demonstrated proportions of CD8+ IFN-γ+ TILs **(G)** and CD4+ IFN-γ+ TILs **(H)** in three groups of control, spermidine and spermidine+Oligomycin under OKT3 stimulation.
