# Supplementary material for: Spermidine Remodels the Mitochondrial Metabolism of Tumor‐Infiltrating Lymphocytes
Source: J Immunol Res. 2025 Oct 29;2025:7550012. doi: 10.1155/jimr/7550012 (PMC12571991; doi:10.1155/jimr/7550012)
Supplement: Supplementary file 2 — Supporting Information 2 Table S1. Data related to the metabolites according to the metabonomic analysis. [file JIMR-2025-7550012-s001.pdf]

Supplementary Table 1. Data related to the metabolites according to the metabonomic analysis

Index	Metabolites	Control				Spermidine			
		Control#1	Control#2	Control#3	Control#4	Spermidine#1	Spermidine#2	Spermidine#3	Spermidine#4
MEMN001	Asparagine	109879	184852	55262	233752	125640	137660	70023	207210
MEMN002	Succinate/Succinic acid	202439	737778	199981	820792	449640	608990	741190	521660
MEMN003	L-Serine	540346	529822	254165	914129	493490	472200	254920	751690
MEMN004	L-Aspartate	4671776	6967259	5289515	13684158	5040700	5577800	5368300	12880000
MEMN005	L-Threonine	1854579	2545259	900641	3679307	1962400	2240700	1213000	3374100
MEMN006	L-Alanine	17989	31002	9513	40209	17047	33320	12140	29025
MEMN007	Adenine	8056	33288	6662	15539	8652	35422	7225.8	13898
MEMN010	Citrate/Citric acid	773280	841778	511534	657317	849910	1137200	535100	1020800
MEMN011	L-Lactate	7740654	36105185	3074757	23341584	9829400	25533000	3970500	17150000
MEMN012	ADP	49050	145267	25582	101198	42524	130070	19608	93222
MEMN013	Fumarate/Fumaric acid	730477	1943037	460136	1959010	868390	1727800	301810	1686700
MEMN014	Uracil	2075794	702852	837825	1786832	1522300	574050	701070	1378100
MEMN015	Guanosine	9287290	2258148	5209903	9778119	11213000	1630700	4377800	8362100
MEMN016	Thiamine pyrophosphate (TPP)	8971	14299	5909	14244	10360	13355	9150.7	17374
MEMN017	Glucose	646916	303874	712058	1455941	915250	699660	1391900	1235700
MEMN018	Oxaloacetate	5023	4605	5920	5241	6870.5	6145.2	7379.4	5148.8
MEMN019	Methylmalonate	184495	799259	230883	793426	441940	663270	816970	564120
MEMN020	Malate	5195981	14958519	1978252	15193069	5148000	12528000	2308700	11297000
MEMN021	Alpha-Ketoglutaric Acid	116888	100548	66885	165832	119450	89281	85150	158080
MEMN022	L-Glutamate	30565421	39371111	18617476	49641584	30690000	36018000	17143000	49482000
MEMN023	Aconitate	30310	25737	16788	19982	47979	40485	18587	37915
MEMN024	L-Tyrosine	319841	531578	90885	393733	294890	371880	117200	344370
MEMN025	Glyoxylate	40823	41047	39536	44025	37739	48167	39162	48604
MEMN026	NAD+	4347	69816	2105	65063	25721	53616	3716.9	166300
MEMN028	D-Glucose 6-phosphate	3184299	201422	852388	3031089	2055000	765810	659380	2106700
MEMN030	Fructose 1,6-bisphosphat	3698505	3249259	1122718	4022178	1380600	4335900	517400	2526600
MEMN031	D-Fructose 6-phosphate	342224	21371	86899	330455	253330	71683	69497	233660
MEMN032	D-Glucose 1-phosphate	540243	320348	428573	580168	520470	352960	456730	511720
MEMN033	Glycerol 3-phosphate	3964860	1478222	727165	13282178	5003800	3810800	547850	13887000
MEMN034	Phosphoenol pyruvic acid	12132710	1237852	6314854	17795050	10058000	11671000	6408200	16290000
MEMN035	6-Phosphogluconic acid	546271	70435	95752	1540594	472500	235180	92438	1129200
MEMN036	Pyruvic acid	11917	46872	13312	42029	21195	36445	9228.2	39477
MEMN037	D-Erythrose 4-phosphate	125888	10443	34045	127505	75645	28608	28337	88726
MEMN038	sedoheptulose 7-	1715981	406963	652466	1299802	1705100	657860	564120	1663200
MEMN039	D-Ribulose 5-phosphate	3811215	1899852	3956117	4139901	6130400	2467500	5151200	4895500
MEMN041	Isocitric acid	221673	174281	136816	138178	228220	268510	137190	252430
MEMN043	AMP	167383	16334815	103388	5794356	764820	15513000	140250	8730700
MEMN048	UMP	34503	2612296	37973	1290891	123620	2280000	38205	1435900
MEMN051	D-Glutamine	1571121	1498741	877262	3101188	1842200	1725700	1213100	3291200
MEMN053	GlcNAc	508542	10320000	318087	3410099	834600	7089000	292020	4714800
MEMN054	BPG	309963	597119	123000	595653	113390	1128400	69044	819000
MEMN056	ATP	508234	1468222	209097	1535347	460430	1620800	199430	1670800
MEMN057	2-Phospho-D-glyceric	3215981	1262444	1489417	7331386	3742100	6129100	1478500	6628900
MEMN061	Xyluose-5-phosphate	1890748	703526	1572233	1553762	2385100	1066300	1662100	1831500

**MEMN063**	**phosphoryle thanolamine**	3894393	5736519	2120583	6143762	3964000	5076900	2010800	5957800
**MEMN064**	**L-citrulline**	13727	23168	15413	16155	20617	15073	12357	19569
**MEMP001**	**L-Arginine**	1578318	1657704	634204	2387525	1488900	1324100	899610	1571700
**MEMP003**	**Acetyl-CoA**	35473	222652	33428	47646	51153	148810	32060	72724
**MEMP007**	**Leucine**	13386916	19381481	7871942	25526733	14610000	17377000	10773000	21789000
**MEMP008**	**Lysine**	41244860	36372593	32242718	69680198	48889000	45708000	41841000	67493000
**MEMP009**	**L-cysteine**	6670	10224	3105	12681	7436.7	9266.7	4317	10540
